# Tracking development assistance for mental health: time for better data

**DOI:** 10.1093/heapol/czac108

**Published:** 2022-12-08

**Authors:** Valentina Iemmi

**Affiliations:** Department of Health Policy, London School of Economics and Political Science, Houghton Street, London WC2A 2AE, UK

**Keywords:** Mental health, development assistance for health, sustainable financing, low- and middle-income countries

Key messages

- Sustainable mental health financing requires accurate financial data, especially in low- and middle-income countries (LMICs) where most people with mental disorders live but resources are extremely limited.
- Development assistance for mental health (DAMH) constitutes a critical source of funding for mental health in LMICs. However, tracking DAMH is complex, and estimates are inaccurate.
- Four groups of limitations at different stages of the estimation process currently hinder the accuracy of DAMH estimates.
- Several opportunities might be leveraged to improve them. This is crucial to support decision makers at both the global and country levels and to strengthen mental health financing in LMICs.

## Introduction

Sustainable mental health financing requires accurate financial data to support funding decisions in the context of limited resources. This is critical in low- and middle-income countries (LMICs) where most people with mental disorders live but resources are extremely limited (Patel *et al.*, 2018). With LMIC governments under substantial fiscal pressure, mental health constitutes as little as 1.8% of governmental health expenditure (WHO, 2018) and is often supplemented by external resources like development assistance for mental health (DAMH) (Chisholm *et al.*, 2019). DAMH includes financial and in-kind contributions disbursed by international organizations for mental health activities in LMICs (Charlson *et al.*, 2017). While crucial for sustainable financing, tracking external resources such as DAMH is complex and current estimates are inaccurate.

Three data sources are used: Financial Tracking Service (FTS) database by the United Nations Office for the Coordination of Humanitarian Affairs; Creditor Reporting System (CRS) database by the Organisation for Economic Co-operation and Development (OECD) and Development Assistance for Health (DAH) dataset by the Institute for Health Metrics and Evaluation (IHME) (Iemmi, 2019). Currently, the DAH dataset constitutes the most sustainable source: it is not only publicly available and regularly updated but also pre-coded (IHME, 2021). However, it presents several limitations that lead to imprecise estimates. This Commentary aims to contribute to ameliorate DAMH estimations, using the DAH dataset as example. After introducing the dataset, I identify its limitations and opportunities for improvement.

## DAH dataset

The DAH dataset reports semi-aggregated disbursements of DAH (Micah *et al.*, 2021). Its structure reflects resource flows from sources (governments, Bill & Melinda Gates Foundation, corporate donations, other private sector contributions, debt repayment and others), through channel organizations, defined as intermediary organizations disbursing funding to LMICs (bilateral and multilateral organizations, multilateral development finance institutions, foundations, nongovernmental organizations and global health initiatives). Annual estimates by health condition, including mental disorders, are available from 1990.

## Limitations

Table 1 summarizes four groups of limitations at different stages of the estimation process: data collection, activity identification, estimation approaches and reporting.

**Table 1.** Limitations and opportunities of IHME DAH dataset for tracking DAMH

	Limitations	Opportunities
Data collection	Few LMIC donors	Repositories (e.g. AidData) and institutional websites of bilateral and multilateral organizations (e.g. BNDES and NDB)
	Few philanthropic donors	OECD dataset on private philanthropy for development
	Few research funders	Dimensions database
Activity identification	Inclusion of some neurological conditions	Exclusion of neurological conditions
	Classification of all disbursements by some organizations under their health issue focus only	Identification of disbursements across all organizations and activities
	Health sector only	Inclusion of sectors beyond health from sources in use (e.g. CRS) and other publicly available sources (e.g. FTS)
Estimation	Adjustments to address under-reporting and reporting lags	Systematic comparison of different approaches (e.g. unadjusted figures)
	Distribution of contributions for projects with multiple focus areas across them	Systematic comparison of different approaches (e.g. upper bounds)
	Country estimates including regional but excluding global and unspecified contributions	Systematic comparison of different approaches (e.g. reallocation of regional, global and unspecified contributions)
Reporting	No disaggregation at activity level	Disaggregation at the activity level when data sources allow (e.g. CRS)
	Philanthropic contributions at source level for BMGF only	OECD dataset on private philanthropy for development

BMGF, Bill & Melinda Gates Foundation; BNDES, Brazilian Development Bank; CRS, Creditor Reporting System; DAMH, development assistance for mental health; FTS, Financial Tracking Service; IHME, Institute for Health Metrics and Evaluation; NDB, New Development Bank; OECD, Organisation for Economic Co-operation and Development.

### Data collection

Data sources used to create the dataset are limited, leading to an underestimation of true figures. Sources include the CRS and Development Assistance Committee databases, International Aid Transparency Initiative database, US Agency for International Development Report of Voluntary Agencies, Internal Revenue Service 990 tax forms, the Foundation Center’s grant database, online grant databases and reports by donors and personal correspondences (Micah *et al.*, 2021). However, they exclude key contributions. LMIC donors are gaining importance in global health, yet only China is currently included (Micah *et al.*, 2019). Similarly, despite the growing role of philanthropy in global mental health, the dataset focuses predominantly on US foundations (Iemmi, 2020). In addition, while private research funders’ interest in the issue is growing, only a few are included (e.g. Wellcome Trust) (Woelbert *et al.*, 2021).

### Activity identification

Inaccurate identification of mental health activities produces both overestimation and underestimation. Project titles and descriptions are searched using mental health-related keywords in nine languages. The search strategy may inflate estimates using terms not only for mental disorders (including substance use disorders, dementia and self-harm) but also for some neurological conditions (epilepsy, headache disorders and Parkinson’s disease) (Micah *et al.*, 2021). Conversely, estimates are conservative for some organizations and activities. Disbursements from global health initiatives and some multilateral governmental organizations (the United Nations Children’s Fund and the Joint United Nations Programme on HIV and AIDS) are classified under the organizations’ health issue focus, though programmes may also include mental health components. Contributions at the health system level that are not exclusively earmarked but that may benefit mental health are not included in final estimates. In addition, despite the increasing integration of mental health activities across sectors and issues, the dataset focuses on health sector only (Iemmi, 2021a).

### Estimation approaches

Poor reporting by donors causes missing data and a reliance on statistical models for estimations. To address under-reporting and reporting lags for earlier years, figures are adjusted using CRS commitment and disbursement data, while figures for the most recent 2 years are estimated using statistical models (Micah *et al.*, 2021). Contributions for projects with multiple focus areas are divided across them proportionally to the number of keywords present in the titles and descriptions. To address the poor quality of information on recipients, regional disbursements are reallocated equally across countries in the region and global and unspecified funding are reported separately.

### Reporting

Limited disaggregation of estimates hampers their use in research and policy. The dataset does not provide disaggregation at the activity level, which could permit a more granular understanding of disbursements, particularly vis-à-vis different mental disorders and population groups (Micah *et al.*, 2021). Individual philanthropic contributions at the source level are available for the Bill & Melinda Gates Foundation only; additional organization names can be accessed upon request, yet only at channel level and only for US foundations (Iemmi, 2020).

## Towards better estimates

Several opportunities could be harnessed to improve estimates across the four stages (Table 1). The first stage is data collection. Better data for LMIC donors are becoming available in repositories (e.g. AidData) (AidData, 2021) and institutional websites of bilateral and multilateral organizations (e.g. the Brazilian Development Bank and the New Development Bank) (BNDES, 2021; NDB, 2021). The OECD dataset on private philanthropy for development, now included in the CRS database, constitutes a promising source for philanthropy worldwide (OECD, 2019). Similarly, contributions by private research funders might be harnessed from the Dimensions database (Digital Science, 2021) reporting recently enhanced mental health figures (Woelbert *et al.*, 2021).

The second is activity identification. While the inclusion of neurological conditions reflects prior conceptualization of the issue (WHO, 2008), their exclusion might better align with more recent approaches (Feigin *et al.*, 2017). Capturing disbursements across all organizations and activities might better reflect the rising approach to mental health support integrated across other health conditions, sectors and issues (Iemmi, 2021a). Similarly, data beyond the health sector might be included from sources already used by IHME (e.g. CRS database) (OECD, 2019) and other publicly available databases (e.g. FTS) (UNOCHA, 2017).

The third stage is estimation approaches. While donors should improve data reporting, systematic comparison of different estimation approaches might illuminate their accuracy and precision (Pitt *et al.*, 2018). For instance, other DAMH datasets use unadjusted figures, total amount disbursed for projects focusing on multiple health areas and reallocation of regional, global and unspecified contributions to recipient countries (Gilbert *et al.*, 2015). Fourth is reporting. Although detailed project descriptions are not available for all disbursements due to poor data or confidentiality issues, activity-level estimates might be reported for some of the included sources (e.g. CRS database). In addition, the OECD dataset on private philanthropy for development represents a promising fount to improve reporting at source level.

## Conclusion

Accurate financial data are crucial to sustainable mental health financing in LMICs. With mental health needs expected to increase in LMICs partly driven by the coronavirus disease pandemic and its policy response (Patel *et al.*, 2018; Santomauro *et al.*, 2021), improving DAMH estimates is urgent to support decision makers at both global and country levels (Shiffman and Shawar, 2020). Current estimates are inaccurate due to four groups of limitations, which reflect customary challenges and trade-offs in producing financial figures from secondary data collected across different organizations and over time, such as limited resources, different classifications and confidentiality issues. This Commentary identifies opportunities to enhance their accuracy at different stages of the estimation process. It is time for better data.

## Data availability

No new data were generated in support of this research.

## Reflexivity statement

The author is a female researcher with over 10 years of experience in mental health policy research, a non-native English speaker and affiliated with a university in a high-income country. She has substantial experience in the quantitative analysis of DAMH, including country-level panel data linkage and analyses (e.g. Iemmi, 2021b).
